# Foreign Medical Teams in the Philippines after Typhoon Haiyan 2013 - Who Were They, When Did They Arrive and What Did They Do?

**DOI:** 10.1371/currents.dis.0cadd59590724486bffe9a0340b3e718

**Published:** 2015-05-05

**Authors:** Kim Brolin, Omar Hawajri, Johan von Schreeb

**Affiliations:** Department of Public Health Sciences, Karolinska Institutet, Stockholm, Sweden; Centre for Research on Healthcare in Disasters, Health System and Policy Research Group, Department of Public Health Sciences, Karolinska Institutet, Stockholm, Sweden; Department of Public Health Sciences, Health system and policy, Karolinska Institute, Stockholm, Sweden

## Abstract

Background: Foreign medical teams (FMT) are international medical teams sent to provide assistance in the aftermath of a disaster. In the last decade, there has been an increase in FMTs deployed following disasters. Despite the potential benefit FMTs might have in substituting the collapsed health care and caring for excess morbidity after large-scale disasters, several studies have demonstrated the difficulties in determining the quality of the response, mainly due to lack of reliable data. In order to bridge the knowledge gap on functioning of FMTs, the aim of this study is to assess the timing, capacities and activities of FMTs deployed to the Philippines after typhoon Haiyan.

Methods: This is a retrospective, descriptive study. Data on characteristics of FMTs present in the Philippines after typhoon Haiyan was provided by the World Health Organization (WHO) and compiled into a single database. Additional data was collected through a web survey, email correspondence and internet searches.

Results: A total of 108 FMTs were identified as arriving to the Philippines within the first month following typhoon Haiyan. None of these were operational in the affected areas within the first 72 h and the average time between arriving and being on-site operational was three days. Of the 108 FMTs, 70% were FMT type 1, 11% were FMT type 2 and 3% were FMT type 3. 16% of FMTs had unknown status. The total number of staff within all these FMTs were 2121, of which 210 were medical doctors, 250 nurses and 6 midwifes. Compared to previous sudden onset disasters, this study found no improvement in data sharing.

## INTRODUCTION

A disaster is a serious disruption of the functioning of a community or a society involving widespread human, material, economic or environmental losses and impacts, exceeding the abilitiy of the affected community or society to cope using its own resources. Sudden onset disasters (SODs) occur with little or no warning, meaning there is insufficient time for the complete evacuation of the at-risk population^1^. Foreign medical teams (FMTs) are groups of health professionals and supporting staff outside their country of origin, aiming to provide health care specifically to disaster affected populations^1^. In recent years, the number of FMTs deployed to SODs has increased^2^ ^3^. Following the 2010 Haiti earthquake, more than 390 FMTs were registered with the WHO Health Cluster, out of which 44 provided inpatient surgical care 2 ^,^ 4. Although FMTs care for injured and sometimes substitute collapsed health systems, questions and concerns related to their work have been raised 2 ^,^ 3. FMTs deployed to SODs have been found to arrive more than 72 hours after the SOD onset and thus too late to care for the most urgent trauma cases 5. Furthermore, FMTs have been criticized for too much focus on trauma care and to be ill adapted to the normal health care needs of the affected area 2 ^,^ 3 ^,^ 6. In light of these concerns, a FMT Working Group was created in order to improve the use of FMTs in SODs 7 ^,^ 8. In 2013, the group finalized the document “Classification and Minimum Standards of FMTs in SODs”, which was endorsed and published by the WHO and Global Health Cluster. This document classified FMTs into three distinct categories: FMT type 1 provides outpatient initial emergency care of injuries and other significant healthcare needs. FMT type 2 provides inpatient acute and surgical care for trauma and other major conditions, while FMT typ 3 provides complex inpatient referral surgical care including critical care capacity 1. The classification was rapidly used and found helpful to define and document the FMTs deployed to the typhoon Haiyan and the 2015 Vanuatu typhoon.

According to a recent UN report, the number of SODs in the Asia-Pacific (AsPac) region has increased during the past years and in 2013, 137 SODs affected the region compared to 93 in 2012 9 ^,^ 10. SODs may create a vast array of effects 11 ^,^ 12, which risk to exceed the relief capacities of the affected country and thereby calling for FMTs to be deployed, particularly in settings where resources are limited 13.

The Philippines is a lower-middle income country located in the AsPac region, with an estimated population of over 98 million people. The country is one of five nations most frequently hit by SODs in the world. In November 2013, the country was affected by typhoon Haiyan 14. This category five typhoon (locally named “Yolanda”) was the most powerful storm ever recorded and the deadliest event of 2013 15 ^,^ 16. More than 6000 people died and over 14 million people were affected, in addition to collapse of infrastructure, health facilities and destruction of livelihoods 15 ^,^ 17 ^-^ 20. Despite a high level of national preparedness including an early warning system and a well-developed disaster response system 18 ^,^ 21, the destruction caused by the typhoon made the Secretary of Health of the Philippines request the immediate deployment of medical teams and emergency supplies to the affected areas 14. According to official reports from the Department of Health (DOH), several domestic teams and FMTs were deployed to the affected region 20 ^,^ 22. Two published papers describe the experiences from individual FMTs in the aftermath of typhoon Haiyan 23 ^,^ 24, but there is to our knowledge no study that systematically has attempted to compile the overall FMT response. The aim of this study is to assess the timing, capacity and activities of FMTs deployed to the Philippines in the first month following typhoon Haiyan.

## METHODS

This is a retrospective descriptive study. Primary and secondary data was collected between 8th of November 2013 and 30th of April 2014. We collected data on: a) Type of FMT (based on the WHO classification level 1, 2, or 3 in addition to number of staff), b) Timing (arrival in country, on site and when the FMT was operational and time of departure) and c) Activities (surgeries, in-patients, out-patients etc) in addition to other data of operational nature.

**Data collection**

*Secondary data review*

To collect all available information on the FMT response to Typhoon Haiyan, we performed an internet search and used additional sources of information, including contacting the study authors for clarifications and more thourugh and detailed information. The search was done using search engines including Google and PubMed. Search words were in various combinations: Typhoon Haiyan/Yolanda, Foreign Medical Teams, Foreign Field Hospitals, Philippines, FMT, Humanitarian Assistance, Sudden-onset disasters, SOD.

*Key informants*

We contacted key persons representing the WHO in the Philippines and asked for data on FMTs deployed after typhoon Haiyan. Extensive information concerning the response was retrieved.

*Web survey*

In a second step, when email or telephone numbers were available, all potential deployed FMTs were invited to join a websurvey, designed using SurveyMonkey® (Appendix 1). Questions concerned period of deployment, key service provision, number of minor and major surgeries, number of outpatients and inpatients per day and the FMT’s classification based on WHO standards^1^. Survey invitations were sent to a total of 96 agencies and was open between 9 May 2014 and June 1, 2014. Reminder emails were made to non-responders two weeks after the first invitation.

**Data compilation**

The results from our search and survey were reviewed, compiled, and entered into a single database using excel. Duplicate information was removed.

## RESULTS

The documents provided by the WHO as well as the Internet searches identified a total number of 151 FMTs deployed to the Philippines out of which 108 arrived to the country during the first month of the typhoon. We were able to contact a total of 96 of these FMTs and these were subsequently invited to partake in the web survey. Of these, 12 (12.5%) responded.

**Type**

Data on classification was obtained through the WHO and the Health Cluster and was available for 91 of the 108 FMTs on site during the first month (Appendix 2). 76 of these FMTs were according to WHO classified as type 1 (Figure 1). These reported a total of 1292 staff, which gives an average of 17 staff per FMT (Appendix 2). 12 of the FMTs were classified as level 2 (Figure 1), with a total of 514 staff, an average of 43 staff per team (Appendix 2). Three FMTs were classified as type 3 (Figure 1). Staff data was available for two of these; one reported the number of staff to be 70 and the other 106 (Appendix 2). For 17 FMTs, classification data was unavailable (Figure 1). These 17 teams reported a total of 131 staff (Appendix 2).

**The number of each type of FMT deployed to the Philippines after typhoon Haiyan.**
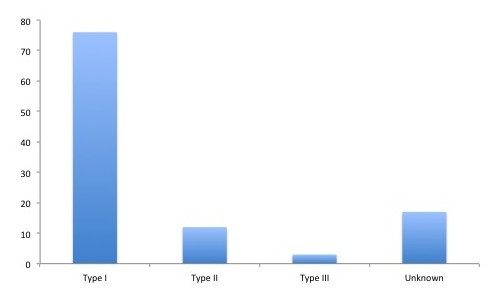
Type I (76), type II (12), type III (3) and unknown category (17).

**Timing**

Data on timing was available for the majority of FMTs. The average time from arrival in the Philippines to the commencement of operational activities on-site was three days. In total, 103 FMTs were operational on-site during the first month after the disaster (Figure 2). We were unable to determine when 45 of the FMTs (30%) became operational. It took up to day 22 post-typhoon before the peak number of FMTs was operational. For 30% of the FMTs, it took more than three days to become operational on-site after their arrival to the Philippines. At total of 45 out of 108 FMTs left the affected areas within the first month (Figure 2). In total, 151 FMTs provided medical support in the affected areas after typhoon Haiyan (Figure 2).

**Timing characteristics of all FMTs deployed to the Philippines after typhoon Haiyan:**
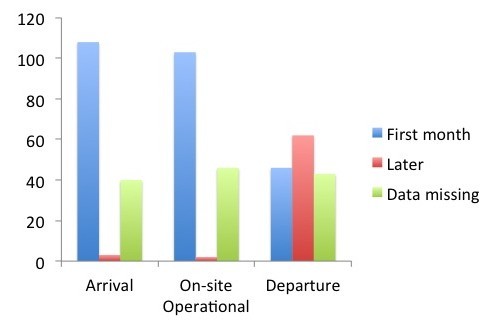
Figure is showing time of arrival, time of being on-site operational and time of departure.

**Activities**

In this study, the total numerical data of inpatient and outpatient services provided by FMTs was quantified by total active days (81). Results are shown in table 1 and represent the 49 FMTs that submitted an exit questionnaire. The total number of minor surgeries performed by FMTs was 4,350, the equivalent of approximately 54 per day. The total number of major surgeries was 490, about six major surgeries conducted daily. The total number of consultations provided within this time was 94934, equaling 1172 daily consultations by all FMT types. The number of inpatient admissions was 2,270, about 28 patients per day. The total number of deliveries was 640 and out of these, six were Caesarean section (Table 1).

**Figure:**
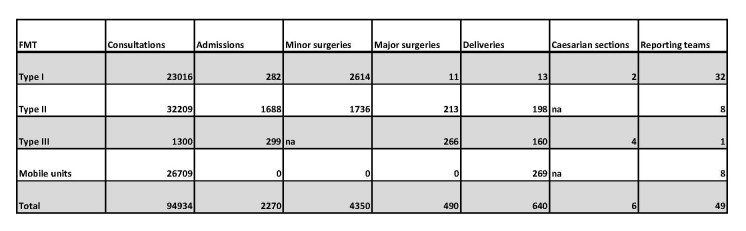
**Table 1. Activities of FMTs in the Philippines after typhoon Haiyan**

## DISCUSSION

Our results show that a significant number of FMTs were deployed to the Philippines albeit; none of them were operational before day three. Apart from data provided by the WHO, it was rather difficult to find robust and reliable data on FMT types, their timing and activities. Only 12% of the contacted teams were ready to share their characteristics and data and partake in the survey. Yet, this is not surprising, as similar findings have been noted in previous studies on FMT response 2 3. However, However, for the sake of accountability, it is necessary for FMTs to systematically and openly report on their activities, not only to researchers but primarily to the Ministries of Health in the countries to which they are deployed. Sharing data and adhering to standards is essential components of the FMT registration, which WHO has set up as a service for affected countries to determine what FMTs they shall accept. Had it not been for the hard work of the WHO in collecting this data and their willingness to share it with us, this study would not have rendered sufficient results to allow publishing. The WHO document on FMT classification and standards was published just before the typhoon and key persons in the WHO office in Manila made systematically use of this, enabling us to know the type of FMT that arrived. This first test of the classification thereby turned out to be positive and for the first time, a systematic classification of FMTs was done and proved to be useful. Following the typhoon that hit Vanuatu in March 2015 the classification was useful to define what type of FMTs that were needed and allow tasking of the 20 FMTs that arrived.

Reasons behind the relative slow set up of FMTs in typhoon-affected areas include difficulties in accessing the hardest-hit archipelagos, lack of transportation in addition to limited information on where the needs were greatest. Initially, many FMTs set out for Tacloban, the city that was struck by a storm surge, which killed a large part of the victims. The dramatic images that were transmitted attracted attention and it was early on “over crowded” with FMTs, while other areas were not accounted for. Two FMTs were only on-site operational for less than five days. These brief missions raise the question of cost-effectiveness and medical value of such short interventions.

Some FMTs that arrived after the first 72 hours demonstrated their ability to deploy ‘fast and light’ and were able to provide emergency and advanced health services for affected populations quickly, although they subsequently faced serious personal and logistical challenges. Lack of logistical support and appropriate training as well as language barriers were reported among FMTs during the response to typhoon Haiyan. Many teams had not fully considered the requirements for full self-reliance and scarcity of data has made it impossible to assess the response as a whole. Agencies reported the number of operations rather than people who underwent surgery.

FMT reports are focused on output data such as number of inpatients and outpatient visits. This does not correspond to people treated but consultations made, meaning that one person can account for many visits. Still, it is often reported as “treating thousands of injured”. Furthermore, surgeries, Caesarian sections and admissions were also reported amongst FMT level 1, despite that inpatient and surgical activity is not applicable for this level^1^. To what extent this is due to FMT mis-classification or that level 1 FMTs actually did provide surgery as an outpatient service despite their classification is not known.

We have not been able to assess to what extent the FMTs deployed were adequately equipped and prepared for the type of health care needs dominating after a typhoon. In lower middle-income countries, non-communicable diseases are increasingly dominating the clinical picture. In addition, the Philippines represent a very different context compared to Haiti for example, having more than four times as many doctors per capita pre-disaster. Also, the burden of disease after a typhoon significantly differs compared to that of an earthquake. WHO reported that minor injuries, including infected wounds, upper respiratory tract infections and chronic diseases such as hypertension dominated the diseases seen in the first month after typhoon Haiyan. The need for trauma surgeons was limited, while only a smaller number of FMT GPs and nurses were needed to relieve the courageous and hard working Philippines doctors and nurses. Thus the main role of FMTs in this setting was to compensate for the collapsed infrastructure and care for “normal conditions”. The fact that 70% of the FMTs deployed were type 1 indicate a relative well balanced response in terms of focus on outpatients rather than on inpatient trauma care.

## LIMITATIONS OF THE STUDY

This study has several limitations out of which many have been mentioned already. The lack of systematic reporting, including lack of defined indicators and regular reporting has been identified as a significant problem. This limits the value of the conclusions from this study. The study design was basic but building on our experience from similar studies. While we are aware of the study limitations, we conclude that despite extensive efforts, it was not possible to retrieve more or better data. We invite colleagues to challenge our results by sharing and compiling more data. We also need to come up with a clear reporting format for deployed FMTs and their actions. Data to be included has been suggested but need to be simple and part of routine data^25^.

## CONCLUSIONS

More than 100 FMTs were deployed to the Philippines during the fist month following the typhoon, but to date it is still not possible to fully assess neither their activities nor their performance. The WHO FMT classification and standards was useful in classifying incoming FMTs while adherence to standards was difficult to monitor. Better, transparent and consistent FMT reporting is needed. We sadly conclude that despite hard efforts and work and significant WHO support we were not able to fully answer our research aim. The WHO-led process to register FMTs is a promising initiative to improve accountability and transparency of FMTs and a tool for affected countries to determine which FMTs to allow in the country. It is clear that an increased evidence-based knowledge is needed to improve and optimize all types of medical disaster response in the future. We need to include more knowledge to direct the good intentioned ambition to help.

## APPENDIX 1: WEBSURVEY (SURVEYMONKEY®)

1 What is the name of your organization?

2 When did your foreign medical team (FMT) arrive in the Philippines?

3 When did your FMT depart from the Philippines?

4 What date was your FMT fully operational?

5 What key services did your FMT provide?

6 How many minor surgeries did your FMT perform per day?

7 How many major surgeries did your FMT perform per day?

8 How many outpatients did your FMT see per day?

9 What was the inpatient capacity of your FMT?

10 What was the classification (Level 1, 2 or 3 according to FMT classification) of your FMT?

**Explanatory Note/ Definitions:**

**Date of Arrival:** The date when the foreign medical team (FMT) arrived to affected areas.

**Date of Departure:** The date when the FMT left affected areas.

**Date of start of on-site operation:** The date when onsite operation was started, i.e. when regular activities started.

**Minor Surgery:** Any surgical procedure that does not involve anaesthesia or respiratory assistance during the surgical procedures. This could involve use of local or regional anaesthesia.

**Major Surgery:** Any surgical procedures that involve general anaesthesia or respiratory assistance.

**Outpatient capacity:** The maximum number of outpatients that the medical team saw per day.

**Inpatient capacity:** The maximum number of inpatients that were hospitalized at the same time. (i.e. bed numbers).

**List of services offered:** The services and functions that are provided by the medical team, also including whether field facility is provided or not.

## APPENDIX 2. FMTs in the Philippines after typhoon Haiyan

Download PDF
